# Symmetry breakdown of electron emission in extreme ultraviolet photoionization of argon

**DOI:** 10.1038/s41467-018-07152-7

**Published:** 2018-11-07

**Authors:** M. Ilchen, G. Hartmann, E. V. Gryzlova, A. Achner, E. Allaria, A. Beckmann, M. Braune, J. Buck, C. Callegari, R. N. Coffee, R. Cucini, M. Danailov, A. De Fanis, A. Demidovich, E. Ferrari, P. Finetti, L. Glaser, A. Knie, A. O. Lindahl, O. Plekan, N. Mahne, T. Mazza, L. Raimondi, E. Roussel, F. Scholz, J. Seltmann, I. Shevchuk, C. Svetina, P. Walter, M. Zangrando, J. Viefhaus, A. N. Grum-Grzhimailo, M. Meyer

**Affiliations:** 1 European XFEL GmbH, Holzkoppel 4, 22869 Schenefeld, Germany; 2 Institut für Physik, University of Kassel, Heinrich-Plett-Straße 40, 34132 Kassel, Germany; 3 Deutsches Elektronen-Synchrotron (DESY), Notkestraße 85, 22607 Hamburg, Germany; 4 Skobeltsyn Institute of Nuclear Physics, Lomonosov Moscow State University, Moscow, 119991 Russia; 5 Elettra-Sincrotrone Trieste SCpA, I-34149 Trieste, Italy; 6 X-Spectrum GmbH, Notkestraße 85, 22607 Hamburg, Germany; 7 SLAC National Accelerator Laboratory, 2575 Sand Hill Road, Menlo Park, CA 94025 USA; 8 Particle Accelerator Physics Laboratory, École Polytechnique Fédérale de Lausanne, 1015 Lausanne, Switzerland; 9 Qamcom Research & Technology AB, Falkenbergsgatan 3, SE-412 85 Gothenburg, Sweden; 10 Paul Scherrer Institut, 5232 Villingen PSI, Switzerland; 11 CNR, IOM, Lab Nazl TASC, I-34149 Trieste, Italy

## Abstract

Short wavelength free-electron lasers (FELs), providing pulses of ultrahigh photon intensity, have revolutionized spectroscopy on ionic targets. Their exceptional photon flux enables multiple photon absorptions within a single femtosecond pulse, which in turn allows for deep insights into the photoionization process itself as well as into evolving ionic states of a target. Here we employ ultraintense pulses from the FEL FERMI to spectroscopically investigate the sequential emission of electrons from gaseous, atomic argon in the neutral as well as the ionic ground state. A pronounced forward-backward symmetry breaking of the angularly resolved emission patterns with respect to the light propagation direction is experimentally observed and theoretically explained for the region of the Cooper minimum, where the asymmetry of electron emission is strongly enhanced. These findings aim to originate a better understanding of the fundamentals of photon momentum transfer in ionic matter.

## Abstract (web summary)

Exploring the photoionization process leads to better understanding of the fundamental interactions between light and matter. Here the authors show the non-dipole contribution in the form of asymmetric photoelectron angular distribution from the ionization of argon atoms and ions.

## Introduction

In photoionization, it is typically assumed that the linear momentum of photons for wavelengths much larger than the size of the absorbing target can be neglected. This is the so-called dipole approximation, which constitutes one of the common approaches to describe light-matter interaction in the wavelength regime up to the extreme-ultraviolet (XUV). However, at very high intensities or at shorter wavelengths the photon’s linear momentum has been shown to become essential for an accurate description of the electron emission. The momentum transfer can lead to a symmetry breakdown in terms of a forward-backward asymmetry in the photoelectron angular distribution (PAD). Generally, the importance of this nondipole effect and its strong influence on various phenomena were demonstrated in numerous studies ranging from fundamental research in atoms and molecules^1,2^, over applications in condensed matter^3^ and realization of high-harmonic generation lasers^4^ to investigations of astrophysical interest^5^. With the rapid evolution of ultrabright X-ray light sources such as free-electron lasers (FELs), the relevance of nondipole effects for photoionization of ionic targets in nonlinear and time-resolved studies gains further importance.

Recent investigations with optical lasers have demonstrated a breakdown of the dipole approximation under strong-field conditions in the long-wavelength regime^6–8^. Possible sources of dipole violations at longer wavelengths are summarized in ref. ^7^ and are mainly related to the relativistic regime for high intensities, to radiation pressure, and magnetic field displacement. Furthermore, in the hard X-ray regime, the wavelength can naturally not be assumed to be much larger than the target, which implies an upper limit to the dipole approximation. A large area of photon energy to intensity correlation may be defined that is supposedly not affected by nondipole effects, called dipole oasis^7^. However, several nondipole phenomena, for example, autoionization and giant dipole resonances as well as quantum interferences^9–15^, are known to be sources for relevant nondipole signatures in the XUV spectral region (see also reviews^1,2^).

Although a manifestation of nondipole effects in the (total) photoionization cross section has not yet been experimentally demonstrated, it is predicted to be directly influenced by the second and generally higher-order nondipole terms for hard X-rays^16,17^. Additionally, recent theoretical studies on extremely high irradiation levels in the order of 10^21^ W cm^−2^ in the XUV regime have predicted a significant influence of the magnetic field component on population mixing between excited states that is strictly forbidden in the dipole approximation^18^. The discussed Raman-type two-photon transition could have a strong, pulse duration-dependent influence on the total cross section.

A well-known showcase for a symmetry breakdown of PADs in linear (one-photon) photoionization at relatively low photon energies is a Cooper minimum^19,20^. This was convincingly demonstrated, for example, for Xe (5*s*) neutral atomic photoionization^21,22^. A Cooper minimum can be described as an energy-dependent drop in the photoionization cross section when the amplitude of a leading ionization channel vanishes at particular energies due to cancellation within the dipole matrix element of the various components of the wave function^23^. This circumstance can give rise to an enhancement of nondipole effects and has been predicted by theory to yield sizeable nondipole effects also in ionic photoionization at similarly low photon energies^24,25^. Though the nondipole effects may be relatively large in this case, the low photoionization cross section in the Cooper minimum poses a challenge to the experimental feasibility of measuring statistically robust spectra of the ionic target. Highly intense femtosecond XUV pulses are required to compensate the substantial cross-section drop in the neutral as well as the ionic target.

Depending on the actual irradiance of the employed FEL pulses and the chosen target, nonlinear interactions with matter can result in sequential emission of few to tens of electrons from a single atom. Most of the related experiments were employing ion spectrometry to be able to capture the highly charged reaction products^26–32^. Investigations of angularly resolved electron emission patterns can originate an even deeper access to the underlying processes^33–35^. Furthermore, merged X-ray-ion beams for ion yield determination via ion spectroscopy at synchrotron radiation sources have attracted broad attention^36–38^. Currently, only angle-integrated photoionization cross sections of positively charged ions are available^39,40^ with, to our knowledge, only one attempt to open the door into angle-resolved measurements^41,42^. Despite the rapid evolution and broad interest of experimental studies on charged systems at synchrotrons and FELs, asymmetric electron emission patterns were hitherto not considered.

Nondipole effects in the photoionization of ionic species can, however, play a substantial role for a cornerstone of FEL science, which is time-resolved studies, where an X-ray trigger pulse ionizes the target before another probing pulse interrogates the evolving system (pump-probe experiments). In most cases, the interest lies predominantly at low charge states^43,44^. Here the photoionization process for ionic matter needs to be accurately understood, including asymmetric electron emission, as imperative for example in studies of evolving chiral systems. The forward-backward symmetry breaking in the electron emission from a chiral molecule via circularly polarized light, which is the photoelectron circular dichroism, is an effective tool to characterize the chiral properties and dynamics of the target^45–47^. The effect strength typically lies in the order of few percent for non-oriented molecules. Thus, even small nondipole contributions independent of the molecular chirality can be crucial for correct data interpretation.

As introduced above, another cornerstone of FEL science is nonlinear photoionization. Nonlinear nondipole effects can be expected to play a significant role in several cases (refs. ^16,48^ and references therein). The topic is so far, however, scarcely explored and was previously only demonstrated for resonantly enhanced multiphoton ionization with optical lasers^49,50^. The present study is making a first step in the direction of exploring nonlinear nondipole effects at shorter wavelengths using FELs.

In the present study, we experimentally reveal the existence of unexpectedly strong asymmetric emission patterns of electrons from sequentially ionized argon atoms in the vicinity of the respective Cooper minimum, using intense femtosecond XUV pulses from the FEL-1 of FERMI in Italy.

## Results

### Modeling

Argon ions were chosen to experimentally prove the existence of a photon momentum transfer in gaseous ionic matter at relatively low photon energies. For argon, the photoionization of an electron from the 3*p* state into the continuum with *d*-symmetry is the dominating dipole ionization channel. It vanishes in the Cooper minimum at photon energies around 50 eV^24^. The main contribution to asymmetric electron emission is originated by interference between the electric dipole (E1) and the electric quadrupole (E2) ionization amplitudes. The magnetic field component M1, in this case, is small and can be neglected^51^.

**Fig. 1:**
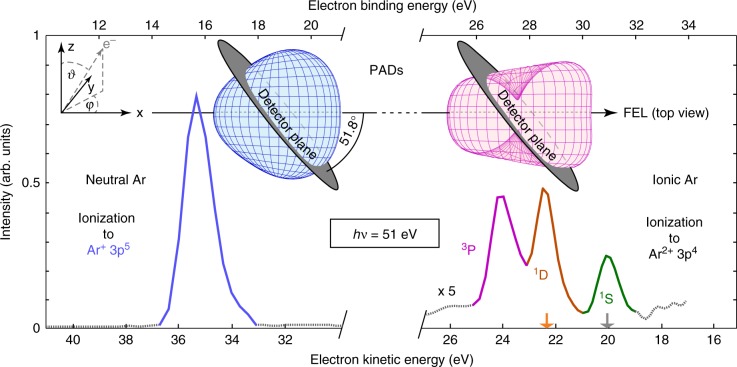
Exemplary electron kinetic energy spectrum and PADs in the Cooper minimum of atomic argon Spectral cut-outs for the first step of ionization into Ar^+^ (3*p*^5^) (left) and two-photon sequential ionization into Ar^2+^ (3*p*^4^) ^1^S (green), ^1^D (red), and ^3^P (purple) ionic states (right) at a photon energy of 51 eV. The intensity of the second ionization step is scaled up by a factor of 5. The orange and gray arrows below the ^1^S and ^1^D indicate overlapping contributions from 3*s* ionization (see text). The depicted spectrum corresponds to only one of the 16 spectrometers at an angle of 45° with respect to the beam propagation for clearer illustration. For the final states Ar^+^ (3*p*^5^) (blue, left) and Ar^2+^ (3*p*^4^) ^3^P (purple, right), the three-dimensional photoelectron angular distribution (PAD) patterns are shown in the upper insets based on the experimental data of all 16 spectrometers (alignment plane shown in the insets, further details in the Methods section) to illustrate the asymmetry of the PADs in the Cooper minimum. The underlying coordinate system is depicted for better orientation (upper left, see also Eq. (1))

Theoretically, the PAD in sequential two-photon double ionization by linearly polarized light, including the first-order nondipole contributions, is of the form

1 $$\begin{array}{*{20}{l}} {\frac{{{\mathrm{d}}\sigma _i}}{{{\mathrm{d}}{\mathrm{\Omega }}_i}}} \hfill & = \hfill & {\frac{{\sigma _i}}{{4\pi }}\left[ {1 + \beta _2^{(i)}P_2({\mathrm{cos}}{\kern 1pt} \vartheta _i) + \beta _4^{(i)}P_4({\mathrm{cos}}{\kern 1pt} \vartheta _i)} \right.} \hfill \\ {} \hfill & {} \hfill & {\left. { + \left( {\delta ^{(i)} + \gamma _2^{(i)}{\kern 1pt} {\mathrm{cos}}^2\vartheta _i + \gamma _4^{(i)}cos^4\vartheta _i} \right){\mathrm{sin}}{\kern 1pt} \vartheta _i{\kern 1pt} {\mathrm{cos}}{\kern 1pt} \varphi _i} \right],} \hfill \end{array}$$

where the indices *i* (*i* = 1, 2) denote the ionization step, *σ* is the corresponding cross section, *β*_2_ and *β*_4_ are the dipole PAD anisotropy parameters, and *γ*_2_, *γ*_4_, and *δ* are the nondipole parameters^24,52^. The spherical angles *ϑ* and *φ* are defined in the coordinate system in Fig. 1 with the *x*-axis along the photon beam propagation direction and the *z*-axis along its polarization vector. Further details about the theoretical modeling can be found in the Methods section.

### Experiment

Ultraintense and ultrashort XUV pulses are required for obtaining statistically robust spectra even at the very low photoionization cross section around the Cooper minima. Such pulses can be produced via an exponential gain of overlapping Bremsstrahlung produced by magnetic chicanes as part of linear electron accelerators^53^. The present experiment was performed at the low density matter (LDM) endstation at the FEL-1 branch of FERMI^54^. The photon energies were chosen to cover the range from 36 to 66 eV. This energy range covers the predicted location of the Cooper minimum of the neutral as well as the singly charged argon^24^. Aiming to increase the total FEL pulse energy to a level that allows for efficient data collection, for most of the reported data, the FEL was operated in a special configuration based on the use of an expanded seed laser pulse. Using the dispersion of a calcium fluoride blade, the seed laser pulse was stretched to about ≈450 fs, resulting in an FEL pulse duration of ≈240 fs, which is triple the typical pulse duration of FERMI FEL-1^55^. Note that in this special case, the FEL pulse length linearly increased with the length of the seed laser without increasing the peak power. Since sequential ionization can happen on comparatively long timescales exceeding hundreds of femtoseconds, the achieved pulse energy of almost 0.5 mJ, even with longer pulse durations, strongly enhanced the sequential ionization rate compared to data recorded with the standard 80 fs and 160 μJ operation mode. To further enhance the maximum irradiance, the FEL pulses were focused by Kirkpatrick-Baez optics^56,57^ to a slightly elliptical spot with a size of ≈10 μm (full width at half maximum), as determined by wavefront sensor measurements. Taking a beamline transmission of about 55% for the chosen energies into account^57^, the argon atoms were irradiated between 5 × 10^14^ and 1.5 × 10^15^ W cm^−2^, which was sufficient for experimentally studying the predicted phenomenon in detail, despite the low photoionization cross section. The photon energy bandwidth was determined to be smaller than 100 meV over the whole energy range of interest, which was beneficial in order to resolve the photoelectrons related to the Ar^2+^ (3*p*^4^) ^1^S, ^1^D, and ^3^P ionic states at binding energies of 31.75, 29.36, and 27.63 eV, respectively^58^ (see spectrum in Fig. 1). Regarding the polarization dependence of the nondipole effect^24,25^, for the present study, we have chosen linear light polarization. The expected high degree of linear polarization was determined with the same experimental setup prior to and during the beamtime to be close to 100%. The measured uncertainty of this value^59^ has negligible influence on the present results.

The photoelectrons emitted from argon under the described conditions were measured with angular resolution, using an array of 16 independently operating time-of-flight spectrometers^60^ including contributions out of the dipole plane. To achieve this expansion of accessible dimensions, the planar spectrometer setup was rotated out of the dipole plane around the *y*-axis by 38.2° (see upper left three-dimensional inset in Fig. 1 and further details in the Methods section). The energy resolution for each of the spectrometers for the present experimental conditions was determined to be *E* (Δ*E*)^−1^ > 200 over the whole energy range of the second ionization step (see spectrum in Fig. 1). The reconstructions of the asymmetric three-dimensional PADs for electron emission from argon at a photon energy of 51 eV for the first and the second steps of ionization are depicted as upper insets in Fig. 1.

**Fig. 2:**
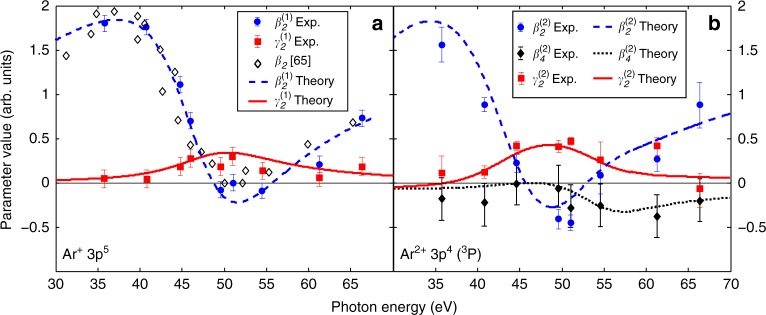
Results for angular distribution parameters. Experimental (symbols) and theoretical (lines) angular distribution parameters of the neutral Ar ionization (**a**) and ionization of Ar^+^ (**b**). The error for the photon energy is given by the FEL bandwidth and jitter, which lies within the symbol size. The parameter errors are determined by accounting for the noise level of the acquisition hardware as well as including the mean fit error of Eq. (1) for 10 intensity bins per photon energy using all 16 spectrometers

For illustration, Fig. 1 shows one representative electron spectrum of a single spectrometer depicting yields of the first ionization step into the Ar^+^ (3*p*^5^) as well as of the second (sequential) ionization step into the Ar^2+^ (3*p*^4^) ^3^P, ^1^D, and ^1^S ionic final states. A sketch of all 16 spectrometers together with their individual yields for the first ionization step of argon in the Cooper minimum is shown in the Methods section. These data points, here as example for illustration, serve as basis for fitting the angular pattern according to Eq. (1). The resulting PAD anisotropy parameters are shown in Fig. 2 together with theoretical calculations. More information about the normalization and calibration of the time-of-flight spectrometers can be found in the Methods section.

## Discussion

Nondipole parameters of neutral argon in the XUV regime were hitherto only calculated theoretically^19,61^. In order to compare the photoionization details from the neutral atom and the singly charged ion, we also present these experimentally determined parameters in the chosen energy range in Fig. 2a. Our present theoretical calculations for the dipole parameter $\beta _2^{(1)}$ and the nondipole parameter $\gamma _2^{(1)}$ are in good agreement with the present data. Due to the presence of a further subsequent photon absorption, counter intuitively, the terms with *β*_4_ and *γ*_4_ [Eq. (1)] can principally contribute even to the PAD of the first ionization step in a two-photon process as discussed in more detail in refs. ^62–64^. However, the contribution is theoretically found and experimentally confirmed to be negligible after summation over the 3*p*^4 3^P, ^1^D, and ^1^S final states of the doubly charged ion. A comparison of the $\beta _2^{(1)}$-values of this first ionization step to previous experimental data of the respective *β*_2_-value from synchrotron measurements^65^ is therefore meaningful and shows good agreement. They are also in accordance with earlier calculations^19,24,61,66^. The observed $\beta _2^{(1)}$ parameter strongly varies from almost maximum positive values (high anisotropy of the PAD) to slightly negative values (close to isotropy) in the neutral atom’s Cooper minimum. The $\gamma _2^{(1)}$ values, representing the nondipole contributions, show a maximum of about 0.3 around 50 eV photon energy.

For the second ionization step in the sequence of the two-photon double ionization, our theoretical approach is based on the stepwise ansatz described in the Methods section and refs. ^52,62^, taking into account the lowest-order nondipole contribution from the interference between electric dipole and electric quadrupole ionization amplitudes. In the present study, we improved our spectroscopic model in comparison to ref. ^24^. This is important because the parameters of the PAD are very sensitive to the atomic model (see Methods for further details). All presented calculations correspond to the velocity gauge. The length gauge gives similar curves, slightly shifted to higher energies. Our theoretical and corresponding experimental results of the dipole and nondipole angular distribution parameters for the sequentially ionized Ar^2+^ (3*p*^4^) ^3^P final state are depicted in Fig. 2b. The ^3^P state was chosen as showcase due to superior statistical validity and the fact that the photoelectron lines from ionization to the other states of Ar^2+^ are energetically overlapping with electron signals of other processes. The Ar^2+^ (3*p*^4^) ^1^D line overlaps with contributions from the singly ionized 3*s* orbital of neutral Ar, and the Ar^2+^ (3*p*^4^) ^1^S with contributions from the ionization of Ar^+^ (3*s*3*p*^6^) to Ar^2+^ (3*s*3*p*^5^) ^3^P (orange and gray arrow in Fig. 1, respectively).

**Fig. 3:**
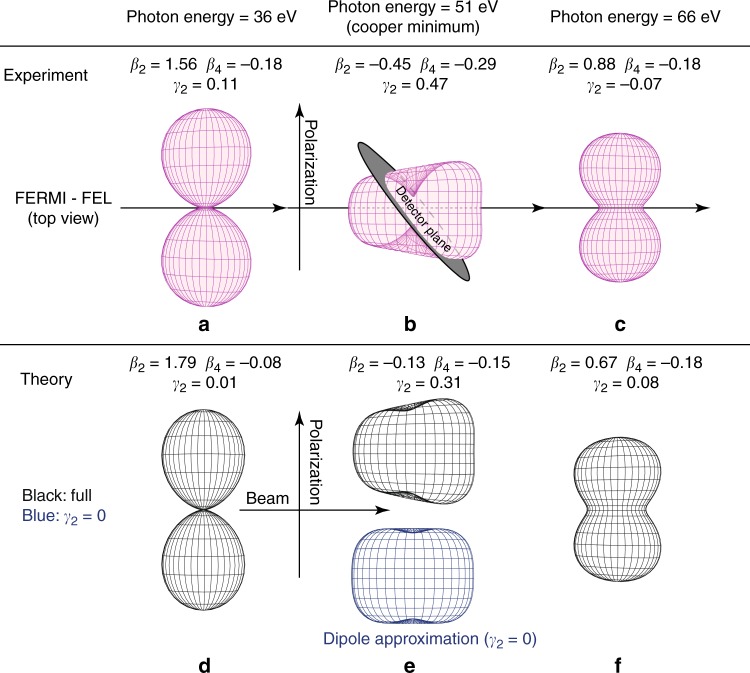
Illustration of angular distributions at different photon energies. Experimentally (**a**–**c**) and theoretically (**d**–**f**) derived PADs at three exemplary photon energies, below (**a**, **d**), in (**b**, **e**), and above (**c**, **f**) the Cooper minimum of Ar^+^ photoionization based on the results presented in Fig. 2. **b** is identical to the inset in Fig. 1 for better orientation. For this case of maximum asymmetry, the experimental PAD is compared to theoretical PADs (**e**) for the cases of including and disregarding the *γ*_2_ contribution. The latter corresponds to the dipole approximation without asymmetric contributions

The $\beta _2^{(2)}$ values reveal a similar behavior as for the first step with strong changes of the PAD in the vicinity of the Cooper minimum in agreement with the general rules given in ref. ^63^. In contrast, the $\beta _4^{(2)}$ values show only small variations and stay always slightly negative. Both experimental dipolar PAD asymmetry parameters are in good agreement with the theoretical calculations. Theoretically, the photon energy for the maximum nondipole effect is located at *hν* ≈ 50 eV for the neutral case and at *hν* ≈ 48 eV for the ionic case (Fig. 2) in agreement with the experimental findings. These relative energy positions, that is the ionic case at a slightly decreased photon energy, also matches the predicted energy relation of the Cooper minima in the photoionization cross section^24^. The maximal strength of the ionic nondipole effect, that is the $\gamma _2^{(2)}$ parameter, is exceeding $\gamma _2^{(1)}$ by more than a factor of 1.5, which is also in good agreement with the present theoretical results. To emphasize the impact of these numbers and illustrate the actual asymmetry, the three-dimensional PAD insets in Fig. 1 are depicting the experiment-based data derived from Eq. (1) in the Cooper minimum at 51 eV photon energy. Note that the left-right asymmetry in these insets corresponds to the *γ*_2_-parameters, whereas the vertical shape is mostly determined by the *β*_2_-parameters. Further experiment-based three-dimensional PADs are shown in Fig. 3 for different photon energies and in comparison to theoretical modeling. Here the influence of the different angular distribution parameters is illustrated, underlining the drastic changes and asymmetries in the Cooper minimum. The overall agreement between experiment and theory can be seen directly. The main difference between the experimentally derived and theoretically calculated PADs in the Cooper minimum (Fig. 3b, e) is the quite large difference in *β*_2_, which is the main reason for the larger vertical constriction in the experimental data. In order to highlight the breakdown of the dipole approximation at *hν* = 51 eV, we show the calculated PADs with *γ*_2_ = 0 and *γ*_2_ = 0.31 (see also Fig. 2) in Fig. 3e.

In previous calculations, the nondipole contribution was underestimated approximately by a factor of two^24^, therefore, it reveals a large sensitivity to the configuration mixing and electron correlations during sequential photoionization. In line with the theoretical predictions^25^, the experimentally determined *δ* and *γ*_4_-parameters (not shown) are close to zero over the whole energy range of this study.

To conclude, we demonstrated that with today’s FELs, the observation of nondipole contributions for (singly) positively charged ions is feasible by means of angle-resolved electron spectroscopy. Our results reveal a strong forward-backward asymmetry of the PAD for the sequential double ionization of argon in the Cooper minima of the neutral atom and the corresponding singly charged ion, therefore providing evidence for the effect of the photon’s linear momentum in both cases. Our study of argon reveals that the effect strength is increased for the photoionization of the singly charged ion. Future studies need to clarify if this is a general phenomenon of ions of different elements and charge states. In order to tackle this question effectively in systematic studies, substantial advances of the available photon sources are required. This may become feasible with high-repetition rate (X)FELs such as European XFEL (Germany) and LCLS II (USA). Those machines are promising to allow for orders of magnitude faster data acquisition whilst meeting and exceeding the current capabilities of (X)FELs. Importantly, the present study opens the door to a more complete description of the photoionization process at high photon intensities, therefore, complementing nondipole studies at, for example, synchrotrons that were so far limited to neutral atoms and molecules. We expect our study to contribute extended perspectives for the investigation of effects of the photon’s linear momentum on (multiple) photoionization as required for for example time-resolved pump-probe studies and for accessing optically forbidden autoionizing states in ions and quadrupole giant resonances. Regarding the latter, further development of our work can potentially bring alternative insights into the features of the giant quadrupole resonance as a possibly combined effect of collapsing f- and g-electron wave functions in the continuum and the collective nature of the ionic shell excitations.

## Methods

### Experiment

The experiment was performed at the LDM endstation of the seeded FEL FERMI FEL-1 in Italy^54^. FERMI delivers ultrashort and ultraintense pulses with a small energy bandwidth, about 100 meV in this case, due to its seeded operation. Six Advanced Planar Polarized Light Emitters undulators of 2.34 m length are employed to create pulses of different duration and intensity, here pulses between 80 and 240 fs with irradiation levels from 5 × 10^14^ to 1.5 × 10^15^ W cm^−2^ with a pulse repetition rate of 10 Hz. The linear polarization used in the experiment is achieved by tuning all six undulators to provide linear horizontally polarized light^59^. For calibration of the spectrometers, both linear horizontal and (right) circularly polarized light were used^59^. For the latter, the quantization axis changes, which allows for an angularly more unified signal in terms of intensity, therefore, allowing for a more robust calibration. Both calibration methods were cross-correlated and included in the error determination. The generated pulses are propagated through the LDM beamline optics with a transmission of approximately 50–60%, depending on the photon energy. A 200 nm Al filter was occasionally inserted for intensity calibration measurements and to clearly distinguish nonlinear from linear contributions. A wavefront sensor was frequently used in order to ensure tight focusing conditions at the respective photon energies. The focal spot was created by mirrors in Kirkpatrick-Baez geometry. Its was measured to be slightly elliptical, revealing energy-dependent sizes from 8 to 12 μm horizontally and from 10 to 15 μm, as taken into account for the theoretical modeling. Especially for the highest intensities, it was important to check that coherence effects in the electron emission into the different ionic final states can be neglected. The photon energy range of the Cooper minimum was mapped from 36 to 66 eV by tuning the FEL to the corresponding harmonic (seventh to thirteenth) of the seed laser (the latter being an optical parametric amplifier set to 243 nm). To cover the energies affected by the Cooper minimum in finer detail, for FEL harmonics 9 and 10, the seed laser wavelength was also set to 250 nm.

**Fig. 4:**
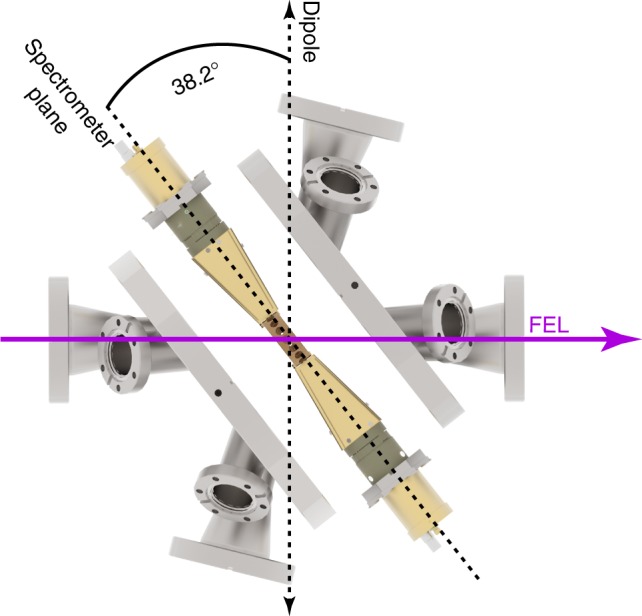
Geometry of spectrometer setup. Drawing of the spectrometer depicting the spectrometer plane as well as the outer vacuum flanges for illustration of the chosen geometry at the LDM beamline at FERMI. The rotation of the spectrometer setup by 38.2° with respect to the dipole plane, or 51.8° with respect to the light propagation, is indicated and can be compared to the geometry shown in Fig. 1

**Fig. 5:**
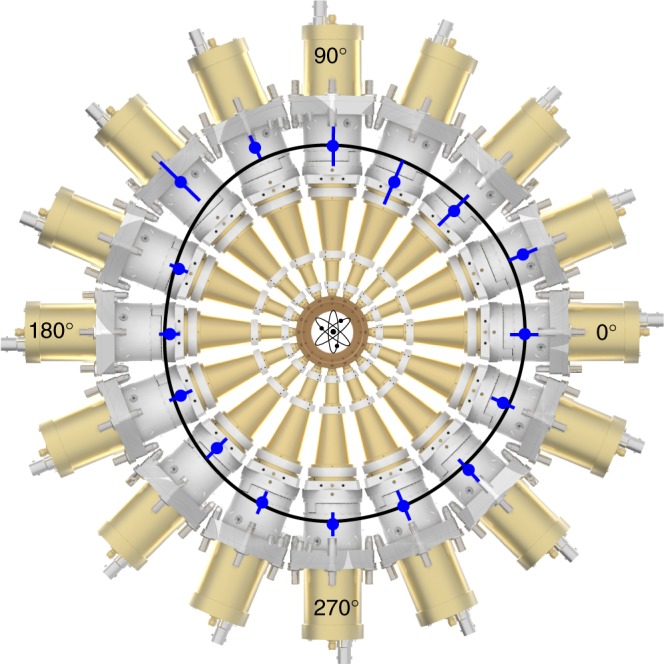
Front view of spectrometer setup with angle-resolved intensity distribution. Front view of the spectrometer setup and its 16 individual devices together with an exemplary angular distribution pattern of neutral argon ionization at 51 eV photon energy. The data points (blue) are transmission-calibrated raw data in the spectrometer plane of the experiment as depicted in Fig. 4, that is 38.2° rotated with respect to the dipole plane. The black curve is a fit based on Eq. (1). The center of the inner ring is the interaction zone where argon atoms are interacting with the FEL photons

The time-of-flight spectrometer setup consisted of 16 individually working spectrometers aligned via rotation around the vertical axis by 38.2°. A detailed sketch of the alignment as well as of the principal spectrometer components with an exemplary angular distribution pattern can be found in Figs. 4, 5, respectively. The microchannelplate (MCP)-based spectrometers are housed in an ultrahigh-vacuum vessel with a typical background pressure of ≈1.5 × 10^−8^ hPa, realized via two 80 l turbomolecular pumps. Prior to the experiment, each MCP-based detector of each spectrometer was adjusted to an operating voltage that ensured linear gain for the experimental conditions. Because of the exceptionally high electron yield for each FEL pulse, up to several hundreds of electrons hit the detector. Hence, the detectors were used in analog mode, that is the time-dependent current through the MCPs was capacitively outcoupled and recorded via analog-to-digital converters (ADCs) at a sample rate of 4 GS s^−1^. The analog signal was furthermore pre-amplified by a factor of ≈15 via broadband amplifiers. The data stream of all 16 ADC channels was then stored into a RAID system. Each single shot of FERMI FEL-1 therefore resulted in 16 raw spectra that were tagged with a bunch ID. This ID allowed for cross-correlation to the machine parameters for each shot, that is intensity and spectrum, being recorded independently.

Argon gas (or neon for calibration measurements (see Data analysis section)) with a purity of 99.995% was injected via an effusive gas jet with a nozzle opening of 100 μm that was aligned to the FEL beam and brought close to the interaction region, that is between 3 and 5 mm. From the light-matter interaction point, the emitted electrons from the argon gas are traveling ≈20 mm before entering the drift tubes. These drift paths are ≈140 mm long, consisting of four conical isolated segments that can individually be operated at different voltages to decelerate the electrons and improve the energy resolution. For the present data, no retardation was applied due to the comparably low electron kinetic energies. The magnetic field resulting from the earth and other surrounding components was compensated via three pairs of Helmholtz coils around the spectrometer setup.

### Data analysis

The rotated spectrometer setup was accounted for according to Eq. (1) in order to extract the angular distribution parameters in the given geometry. Each of the 16 spectrometers was time-to-energy calibrated via separate measurements of Ne 2*p* electrons at different FEL wavelengths. Here the known photon energy in combination with the measured electron time-of-flight through the spectrometer drift tube can be converted into kinetic energy of the photoelectrons via a functional behavior of

2 $$E_{{{h}}\nu } = \frac{1}{2}m_{\mathrm{e}}\left( {\frac{s}{{t - t_0}}} \right)^2 + E_{{\mathrm{Ne2}p}}$$

Here *m*_e_ is the electron mass, *s* is the length of the drift path for each spectrometer, *t* − *t*_0_ the time-of-flight, and *E*_Ne2*p*_ the binding energy of neon 2*p* electrons, that is 21.7 eV. The Ne 2*p* electrons also serve as calibration for the transmission of each single spectrometer since their angular distribution parameter *β*_2_ is well known^67^. By applying the known angular distribution to determine a calibration factor for each spectrometer at different photon energies, a transmission function was derived and consistently applied to all data. The calibration measurements are taken with and without inserted aluminum filter to include a potential alignment of the target by the presence of a second ionization step. The aluminum filter highly suppressed the residuals of the seed laser and reduced the FEL transmission by a factor of ≈0.7. By observing the intensity reduction due to the inserted filter, a cross check for nonlinear contributions was possible. The individual detector response functions were determined by low signal spectra and used for deconvolution. For the error determination, two contributions were taken into account. First, a random intensity binning was applied to the data in order to derive the statistical error. Second, the baseline fluctuation of each spectrometer was used to derive the area error. Both contributions were summed up as basis for the shown errors in Fig. 2.

### Modeling

We consider the sequential 2PDI as a two-step process^62^:

3 $${\mathrm{Step}}\,1: \hbar \omega + {\mathrm{Ar}}\left( {3{{p}}^6} \right) \to {\mathrm{Ar}}^ + \left( {3{{p}}^5\,{}^2{\mathrm{P}}_{1/2,3/2}} \right) + {\mathrm{e}}_1,$$

4 $${\mathrm{Step}}\,2: \hbar \omega + {\mathrm{Ar}}^ + \left( {3{{p}}^5\,{}^2{\mathrm{P}}_{1/2,3/2}} \right) \to {\mathrm{Ar}}^{2 + }\left( {3{{p}}^4\,{}^3{\mathrm{P}}_{0,1,2}} \right) + {\mathrm{e}}_2.$$

The first step leads to the aligned intermediate Ar^+ 2^P_3/2_ state in the case of linearly polarized photons, while the Ar^+ 2^P_1/2_ remains isotropic. As second step, the ion is further ionized by the second photon, with emission of the second electron e_2_. Detailed expressions for the asymmetry parameters defined in Eq. (1) in terms of the ionization amplitudes are given in ref. ^52^. The dominant nondipole contribution comes from the interference of the E1 and E2 photoionization amplitudes, therefore, we neglect the M1 amplitudes. The amplitudes were consistently calculated in the velocity gauge.

The electron wave functions were obtained in a multiconfigurational Hartree-Fock approximation^68^. The wave function for a discrete atomic (ionic) state, characterized by parity *π*, total orbital momentum *L*, spin *S*, and other quantum numbers *α* is expanded in terms of the configuration state functions (CSFs)

5 $${\kern 1pt} \left| {\alpha ,\pi LS} \right\rangle = \mathop {\sum}\limits_{r = 1}^{n_r} {\kern 1pt} c_r(\alpha ){\kern 1pt} \left| {\xi _r,\pi LS} \right\rangle ,$$

where *ξ*_*r*_ denotes the configuration and internal angular momenta couplings, and *n*_*r*_ is the number of CSFs. The mixing coefficients, *c*_*r*_(*α*), are found by diagonalizing the nonrelativistic atomic (ionic) Hamiltonian.

For steps (3) and (4), we use different sets of basis electron functions and CSFs, suited for the corresponding step.

For the second step (4), we started with the self-consistent term-average CSF 3*p*^4^ and generated other ${{n}}\ell$ orbitals by the term-average frozen-core Hartree-Fock ${{3p}}^3n\ell$ calculations. The set of CSFs for the initial ion state Ar^+^ included single and double excitations from the 3*s*^2^3*p*^5 2^P configuration to 3*p*, 4*s*, 3*d*, 4*p* states, [3*s*^2^(3*p*^5^ + 3*p*^4^4*p*) + 3*s*3*p*^5^(4*s* + 3*d*) + 3*s*^2^3*p*^3^(4*s*^2^ + 4*p*^2^ + 3*d*^2^) + 3*s*3*p*^4^(4*s*4*p* + 3*d*4*p*) + 3*p*^5^(3*d*4*s* + 4*p*^2^ + 4*s*^2^ + 3*d*^2^) + 3*p*^6^4*p*] ^2^P, with all orbitals frozen. Frozen-core *LS*-dependent Hartree-Fock functions of ${{E}}\ell$ continua, $\left( {3{{p}}^4 + 3{{p}}^34{{p}}} \right)E\ell ^{2{{S}} + 1}{{L}}$, were taken for the final Ar^2+^ + e_2_ state, where *E* and $\ell$ are the kinetic energy and orbital angular momentum of the photoelectron. *L* and *S* are the total orbital and spin of the final state.

For the first step (3), we used a similar approach. In particular, we started with self-consistent CSF 3*p*^5^ and generated other ${{n}}\ell$ orbitals by the term-average frozen-core ${{3p}}^4n\ell$ calculations. The initial (ground atomic) state was represented by the expansion [3*s*^2^(3*p*^6^ + 3*p*^5^4*p*) + 3*s*3*p*^6^4*s* + 3*s*^2^3*p*^4^(4*s*^2^ + 4*p*^2^ + 3*d*^2^ + 3*d*4*s*) + 3*s*3*p*^5^(4*s*4*p* + 3*d*4*p*) + 3*p*^6^(4*p*^2^ + 4*s*^2^ + 3*d*^2^)] ^1^S. Finally, the frozen-core functions of ${{E}}\ell$ continua, $\left( {3{{p}}^5 + 3{{p}}^44{{p}}} \right)E\ell \,{}^2{\mathrm{P}}$, were taken for the final Ar^+^ + e_1_ state.

The current model can be compared to the previous calculations of the nondipole effects in photoionization of Ar^+^^24^. The main improvements in the present study come from including the configurations Ar^+^ (3s3p^6^) and Ar^2+^ (3s3p^5^) in expansions (5) for the single and doubly charged ion, respectively, and from using basis electron wave functions optimized on the final states of the corresponding step, that is Ar^+^ and Ar^2+^, instead of Ar and Ar^+^, respectively. Except from the two configurations mentioned above, more were included in our model in comparison with^24^, but their influence was not large. The above improvements of the atomic model result in (a) better agreement of the theoretical threshold energies with the experiment and (b) a more accurate relative position of the Cooper minima in neutral Ar and in Ar^+^. Simultaneous artificial implementation of both points by shifting the ionization thresholds (as was done in ref. ^24^) is not possible. The improvement of the model is especially important in our case, where the results are highly sensitive to both, the relative positions of the Cooper minima and to the ionization thresholds. For example, shifting slightly the zero of the 3*p*-Ed partial ionization amplitude, we strongly change the amplitude’s ratio in different ionization channels, causing variations of the anisotropy parameters, which are expressed in terms of these ratios. One can find an example of the sensitivity to the relative positions of Cooper minima in ref. ^25^ for the case of circularly polarized light.

Calculations of the PADs were performed for both intermediate fine structure ionic states Ar^+^ (3*p*^5 2^P_1/2_, ^2^P_3/2_) and for the three fine structure levels of the residual doubly charged ion Ar^2+^ (3*p*^4 3^P_0,1,2_). All numerical results are summed over unresolved fine structure states. Here we assume that the fine structure states of the intermediate ion are excited incoherently.

## Data Availability

The raw and processed data of this study are available upon reasonable request addressed to the corresponding author.
